# Enhancing nutrient intake, egg production, and egg quality by fermented *Leucaena leucocephala* leaf meal in a diet of laying quail

**DOI:** 10.14202/vetworld.2025.133-140

**Published:** 2025-01-22

**Authors:** Merry Muspita Dyah Utami, Abigeil Akbar

**Affiliations:** 1 Department of Animal Science, Politeknik Negeri Jember, Jl. Mastrip PO Box 164, Jember 68121, Indonesia; 2 Laboratory of Feed Technology, Politeknik Negeri Jember, Jl. Mastrip PO Box 164, Jember 68121, Indonesia

**Keywords:** Egg production, egg quality, fermentation, *Leucaena leucocephala*, nutrient intake

## Abstract

**Background and Aim:**

The inclusion of *Leucaena leucocephala* leaf meal (LLM) in poultry feed is often limited due to its high crude fiber and mimosine content. This study investigates the potential of fermented LLM (FLM) to enhance nutrient intake, egg production, and egg quality in laying quails by reducing anti-nutritional factors through fermentation.

**Materials and Methods:**

Two hundred 42-day-old laying quails were assigned to five dietary treatments: T0 (control) = 100% basal diet (BD), T1 = 98% BD + 2% FLM, T2 = 96% BD + 4% FLM, T3 = 94% BD + 6% FLM, and T4 = 92% BD + 8% FLM. Parameters including nutrient intake (energy, protein, fat, calcium, phosphorus), feed conversion ratio (FCR), egg production, egg weight, eggshell thickness, Haugh unit, and yolk color were measured over a 28-day trial. Data were analyzed using a one-way analysis of variance, followed by Duncan’s test for significant differences (p ≤ 0.05).

**Results:**

FLM supplementation significantly improved energy, protein, fat, calcium, and phosphorus intake while reducing FCR. At 4% FLM inclusion, significant enhancements in egg production, eggshell thickness, Haugh unit, and yolk color were observed. Conversely, fiber intake and overall feed intake remained unaffected across treatments. The highest egg production (56.43%) and best yolk color (8,95) were observed in the 8% FLM group.

**Conclusion:**

Incorporating FLM into the diets of laying quails effectively enhances nutrient utilization and improves egg production and quality without increasing feed intake. The optimal inclusion level for maximizing benefits appears to be 4-8% FLM.

## INTRODUCTION

The quail is a bird with considerable potential as a source of animal protein [1]. It has been observed that quail are capable of producing 290–300 eggs/year. To reduce feed costs, alternative feed ingredients are used as substitutes for conventional feed [2]. *Leucaena leucocephala* is a strategic source of protein for poultry and can be used as an alternative feed ingredient, particularly in tropical regions [3, 4]. The availability of *L. leucocephala* does not compete with human consumption, which can reduce the feed costs of laying quail [5]. The proximate composition of *L. leucocephala* leaf, which contains 8.7% ash, 4.1% ether extract, 21.9% crude protein, 19.1% hemicellulose, 17% cellulose, and 5.7% lignin [6]. In addition, tannins were at 4.1%, and mimosine was at 6.77% [7]. It also contains carotenoids and xanthophylls, which are the primary precursors of egg yolk pigmentation [3]. The use of *L. leucocephala* leaves is limited because of its high crude fiber content and the presence of the anti-nutrient compound mimosine [8]. The crude fiber content of the plant material can reach 30%, which exceeds the maximum permitted level of 6% for inclusion in quail feed [9]. The crude fiber consists of cellulose and hemicellulose [10]. The digestive tract of poultry lacks the enzymes required to digest cellulose [11]. High fiber intake results in a shorter digestive process and reduced digestibility [11–13]. High levels of crude fiber in the diet have been demonstrated to reduce the efficiency with which nutrients are used [11]. In addition, the limitation of *L. leucocephala* is the presence of mimosine and tannin [14]. Consuming mimosine more than 5% of the tolerance limit causes poisoning [15]. The structure of mimosine is similar to that of tyrosine, and chickens consuming *L. leucocephala* leaves will recognize it as an amino acid tyrosine. As a result, the body becomes deficient in the amino acid tyrosine, and the production of the hormone thyroxine is disrupted [16]. Mimosine is an anti-nutrient substance that can interfere with the absorption of nutrients in the digestive tract [17]. Tannin affects fish growth [18]. Tannin is a protease enzyme inhibitor that inhibits protein digestibility in the digestive tract, thereby reducing the palatability of the feed and thereby reducing its intake and growth [19]. Therefore, efforts are needed to reduce the high levels of fiber and anti-nutritional substances.

The toxicological evaluation is an important aspect of research on *L. leucocephala*, and it requires further investigation and resolution. The issue of detoxification remains unresolved, which in turn limits its potential use as a feedstuff [17, 20]. The technology used to reduce crude fiber content and increase protein content while simultaneously reducing mimosine compounds is fermentation [21, 22]. Fermentation is defined as a chemical change in feed ingredients caused by microorganisms [23]. The effective microorganisms (EM)-4 are a solution comprising decomposer microorganisms, including *Lactobacillus* spp., *Streptomyces*, cellulose-degrading fungi, lactic acid bacteria, phosphorus-solubilizing bacteria, and photosynthetic bacteria, which can be utilized for fermentation purposes [24]. The basic principle of the fermentation process is the breakdown of the crude fiber into simple sugars that are readily digestible [25]. Fermentation treatment of *L. leucocephala* leaf meal reduces mimosine and crude fiber levels, preventing any interference with chicken growth.

Numerous previous studies [4, 5, 7, 13, 18, 25] have shown that *L. leucocephala* leaf meal has been used extensively in animal feed. However, its use is limited by the high levels of crude fiber and mimosine in the material. This study aimed to identify an *L. leucocephala* leaf meal that can be used as a protein source for laying quail by removing the limitations of its use through a fermentation process to reduce crude fiber and mimosine content. The novelty aspect of this study was to investigate how the fermentation process increased the nutrient content of *L. leucocephala* leaf meal. The potential benefit and novelty of this study are the approaches to investigate the effect of fermentation on increasing nutrient intake to improve quail egg production and quality.

## MATERIALS AND METHODS

### Ethical approval

All animals used for research were kept following the Commission Recommendation on Guidelines for the Accommodation and Care of Animals Used for Experimental and Other Scientific Purposes [26]. The research methodology, sample collection, and analysis methods were thoroughly reviewed and approved by the Animal Ethics Committee of Politeknik Negeri Jember No. 503/PL17.4/PG.

### Study period and location

The study was conducted from July to December 2022 at the Animal Production and Feed Technology Laboratory, Department of Animal Science, Politeknik Negeri Jember.

### Fermented *L. leucocephala* leaf meal (FLM) preparation

The leaves of *L. leucocephala* were obtained from the Livestock and Forage Nursery in Jember according to the identification given by the International Plant Names Index. Published on the Internet http://www.ipni.org, The Royal Botanic Gardens, Kew, Harvard University Herbaria & Libraries, and Australian National Herbarium. The leaves were separated from the stem, cleaned, and soaked in water for 12 h. They were then dried and ground until they reached a size of 1 mm, thus producing the desired *L. leucocephala* leaf meal, and it was stored at a temperature of 27°C–30°C. The meal was subjected to fermentation using an EM-4 inoculant solution containing *Rhizopus oligosporus*, *Aspergillus niger*, and *Saccharomyces cerevisiae*, which was cultured on potato dextrose agar medium and incubated at 30°C for 24 h. The inoculant solution was then diluted with 100 mL of distilled water. The density was 10^10^ colony-forming units [CFU]/mL [27], which were then subjected to a 30-min steaming process before fermentation to ensure the sterilization of the material from potential microorganisms. Subsequently, 100 mL of the inoculant solution with a density of 10^10^ CFU/mL was added to 1 kg of *L. leucocephala* leaf meal and mixed until a uniform distribution was achieved. The mixture was incubated at 30°C for 24 h. Molasses were added at a 1:1 ratio. The fermentation ratio was 40 mL of EM-4 with 60-g *L. leucocephala* leaf meal [28]. The material was then placed in an anerobic environment for 8 days. Subsequently, the FLM was analyzed to determine its crude protein, crude fiber, ether extract, calcium, phosphorus, and metabolizable energy content.

### Basal diet (BD)

The BD was formulated with the following ingredients: corn, rice bran, fish meal, soy meal, clamshell meal (CSM), and FLM. The ingredient composition of the BD is presented in Table 1, and the ingredient composition of the experimental diet is presented in Table 2.

**Table 1:** Ingredient components of the basal diet.

Component	Metabolizable energy (kcal/kg)	Protein (%)	Fiber (%)	Fat (%)	Calcium (%)	Phosporus (%)
Corn	3300	8.79	3.8	2.5	0.01	0.13
Rice bran	1900	13	5	12	0.06	0.8
Fish meal	2750	55	2	1	6.5	4
Soya meal	2550	42	0,5	3	0.2	0.33
CSM	0	0	0	0	28.25	0.79
FLM	3267	28.1	10.36	14	0.47	2.5

BD=Basal diet, FLM=Fermented *Leucaena leucocephala* leaf meal, CSM: Clamshell meal

**Table 2:** Composition of the experimental diet.

Parameters	100% BD	98% BD + 2% FLM	96% BD + 4% FLM	94% BD + 6% FLM	92% BD + 8% FLM
ME (kcal/kg)	2836	2899	2843	2850	2862
Protein (%)	21.28	21.32	21.33	21.96	21.55
Fiber (%)	2.96	3.13	3.40	3.36	3.54
Ether Extract (%)	3.41	4.12	4.23	3.98	4.22
Calcium (%)	1.61	1.68	1.63	1.70	1.65
Phosphor (%)	0.74	0.78	0.79	0.81	0.78

Analyzed at the Laboratory of Feed Technology, Politeknik Negeri Jember, Indonesia, BD=Basal diet, FLM=Fermented *Leucaena leucocephala* leaf meal

### Experimental design

Two hundred laying hen quails (*Coturnix coturnix japonica*) 35 days old were obtained from the Laboratory of Animal Production, Department of Animal Science, Politeknik Negeri Jember, were randomly divided into five groups and four replications, comprising a sample of 10 quails. The weather conditions in Indonesia from July to December 2022 at the time of this study were 54%–57% and 80% humidity. The study was divided into two periods: adaptation to the treatment diet and data collection. Adaptation to the treatment diet was conducted for 7 days from 35 days to 41 days of age before data collection began. Data collection began at 42 days of age. The groups were as follows: (1) Control = 100% BD + 0% FLM; (2) 98% BD + 2% FLM; (3) 96% BD + 4% FLM; (4) 94% BD + 6% FLM; and (5) 92% BD + 8% FLM. The experimental method involved direct observation of subjects who were provided with diet treatments from 42 days to 70 days of age.

The feed intake data were collected daily during the treatment period. Energy intake was calculated by multiplying the amount of feed provided (in grams) by the energy content (in calories per kilogram), as well as the intake of protein (in grams), fat intake (in grams), fiber intake (in grams), calcium intake (in grams), and phosporus intake (in grams) [29]. The body weight of each quail was calculated as the mean of the weight recorded before and after the treatment period. Feed conversion is the proportion of feed consumed over a specified period relative to the weight of eggs produced during that period [30]. Egg weight was determined by weighing the eggs on an analytical scale [31]. The number of eggs produced is divided by the amount of laying quails to obtain the egg production ratio, which is then multiplied by 100%. The body weight of each quail was calculated from the average body weight before and after the treatment period. Feed conversion is the amount of feed consumed during a given period to the weight of eggs produced during the same period [32]. The eggshell thickness was determined using a screw micrometer [33]. The Haugh unit was calculated by measuring the height of the albumen and the weight of the egg [34]. The hue of the yolk was determined using the Roche egg yolk color fan [35].

### Statistical analysis

The data collected during the study were analyzed using a one-way analysis of variance (ANOVA) to evaluate the effects of different levels of FLM inclusion on nutrient intake, feed conversion ratio (FCR), egg production, and egg quality parameters. Significant differences among treatment means (p ≤ 0.05) were further evaluated using Duncan’s multiple range test. Statistical analyses were conducted using the Statistical Package for the Social Software Version 22 (SPSS, IBM Corp. NY, USA).

The analysis included nutrient intake parameters such as energy, protein, fat, fiber, calcium, and phosphorus. Production performance, including egg production percentage and feed conversion ratio, was also evaluated to assess productivity and feed efficiency. In addition, egg quality parameters, including egg weight, eggshell thickness, Haugh unit, and yolk color, were analyzed to determine the effect of FLM on external and internal egg quality.

Statistical differences were presented as mean ± standard deviation (SD) in tables. Results were deemed statistically significant if treatment means had different superscript letters in the same column (e.g., *a, b*, and *c*), indicating differences at (p ≤ 0.05). Data interpretation followed established standards for poultry performance metrics.

## RESULTS AND DISCUSSION

Data on feed intake, body weight, and feed conversion for each treatment are presented in Table 3. Based on the analysis of variance, there was no significant difference in feed intake, but there were substantial differences in metabolizable energy, protein, fat, calcium, and phosphor intake. There were no significant differences in fiber intake among the treatments. Table 4 presents data on hen day production, feed conversion, egg weight, eggshell thickness, Hough unit, and yolk color.

**Table 3:** Effects of FLM on nutrient intake of quail (per day).

Parameters	100% BD	98% BD + 2% FLM	96% BD + 4% FLM	94% BD + 6% FLM	92% BD + 8% FLM
Feed intake (g)	22.79 ± 0.02	22.81 ± 0.01	22.82 ± 0.02	22.89 ± 0.01	22.92 ± 0.01
Energy intake (cal)	64.64 ± 1.02^a^	68.41 ± 1.24^b^	65.61 ± 1.24^b^	65.25 ± 1.22^b^	65.62 ± 1.27^b^
Protein intake (g)	4.85 ± 0.01^a^	4.92 ± 0.01^b^	4.86 ± 0.01^b^	5.03 ± 0.02^b^	4.94 ± 0.02^b^
Fiber intake (g)	0.67 ± 0.01	0.71 ± 0.01	0.78 ± 0.01	0.77 ± 0.01	0.77 ± 0.01
Fat intake (g)	0.78 ± 0.02^a^	0.94 ± 0.02^b^	0.96 ± 0.03^b^	0.91 ± 0.02^b^	0.97 ± 0.03^b^
Calcium intake (g)	0.368 ± 0.01^a^	0.384 ± 0.01^b^	0.371 ± 0.01^b^	0.390 ± 0.02^b^	0.378 ± 0.01^b^
Phosphorus intake (g)	0.169 ± 0.01^a^	0.178 ± 0.02^b^	0.179 ± 0.02^b^	0.185 ± 0.02^c^	0.179 ± 0.01^b^

Different letters in the same column mean a statistical difference at p *≤* 0.05. BD=Basal diet, FLM=Fermented *Leucaena leucocephala* leaf meal

**Table 4:** Effects of FLM on the production and egg quality of quail.

Parameters	100% BD	98% BD + 2% FLM	96% BD + 4% FLM	94% BD + 6% FLM	92% BD + 8% FLM
Egg production (%)	53.15 ± 0.98^a^	54.54 ± 0.22^b^	54.92 ± 0.18^b^	55.25 ± 0.21^b^	56.43 ± 0.24^c^
Feed conversion (kg)	2.33 ± 0.32^b^	2.28 ± 0.28^a^	2.27 ± 0.26^a^	2.19 ± 0.24^a^	2.12 ± 0.22^a^
Egg weight (g)	9.97 ± 0.82^a^	10.09 ± 0.76^b^	10.66 ± 0.78^b^	10.85 ± 0.71^b^	11.02 ± 0.94^b^
Eggshell thickness (mm)	0.16 ± 0.01^a^	0.17 ± 0.01^b^	0.18 ± 0.01^b^	0.19 ± 0.03^b^	0.19 ± 0.02^b^
Hough unit	58.20 ± 0.14^a^	59.28 ± 0.23^b^	59.71 ± 0.21^b^	59.50 ± 0.20^b^	59.24 ± 0.06^b^
Yolk color	7.53 ± 0.54^a^	7.58 ± 0.35^b^	8.17 ± 0.42^c^	8.23 ± 0.48^c^	8.95 ± 0.56^c^

Different letters in the same column mean a statistical difference at p *≤* 0.05. BD=Basal diet, FLM=Fermented *Leucaena leucocephala* leaf meal

The analysis of feed intake revealed no significant differences between the experimental groups. This is likely because the feed ingredients met the standard nutritional requirements for laying quail, including metabolizable energy at 2700 kcal/kg, crude protein at 17%, fat at 7%, calcium at 0.9%–1.2%, phosphor at 0.6%–1%, ash at 8%, and fiber at 7%. The protein-energy balance remained unchanged across all treatments, resulting in no significant difference in feed intake. The protein-energy balance in feed affects feed intake [36]. A low protein-energy balance was demonstrated to increase feed intake, whereas a high protein-energy balance was shown to decrease feed intake.

The more FLM inclusion in the diet, the more protein is contained in the feed. Leucaena is a source of protein with a protein content of 25.2%–32.5% [37]. Furthermore, the FLM exhibited higher crude fiber content than the control. The use of FLM increases metabolic energy intake because fermentation can break down crude fiber, thereby increasing energy intake [38]. The fermentation process can enhance the nutritional value of ingredients by facilitating the biosynthesis of vitamins, essential amino acids, and proteins while also improving protein quality and reducing crude fiber [38-40]. EM-4 bacteria produce cellulase and xylanase enzymes that degrade cellulose into cellobiose. The end product of this process is glucose. This resulted in fermented feed having superior nutritional value, greater digestibility, and improved absorption [41]. The fermentation enhances the crude protein content [42]. Fermentation can effectively reduce the crude fiber content by approximately 18% [22].

Fermentation increases nutrient intake, as evidenced by increased protein and amino acid intake with increasing FLM. The essential amino acid content of *Leucaena* leaves is arginine 2.20%, histidine 0.74%, isoleucine 2.44%, leucine 3.02%, lysine 2.37%, methionine 0.58%, phenylalanine 1.89%, threonine 1.94%, tryptophan 0.31%, and valine 2.31% [39]. Digestibility indicates the amount of nutrients and energy that can be absorbed and utilized [36, 40]. The intake of nutrients by FLM enhances the amount of nutrients consumed. The higher the digestibility of nutrients, the more energy can be utilized for growth; therefore, even if the energy-protein balance is the same, protein and energy intake will result in higher digestibility than the control [42].

The use of FLM in the diet increases egg production. The metabolic process in poultry is influenced by the digestibility and metabolism of the feed [43]. The FLM affects the metabolic process runs well, resulting in high productivity. Metabolic processes influence production [44]. During the fermentation process, EM4 supplementation of *L. leucocephala* leaf meal supplemented with EM4 effectively reduces mimosine. This is supported by research [22], which found that fermentation reduces mimosine by 51.24%, so it does not affect quail productivity. *L. leucocephal*a leaves contain alkaloids, flavonoids, saponins, tannins, and polyphenols. The metabolites play an important role in exhibiting antioxidant properties as protective agents against radical-mediated disease processes, these connections function as antioxidants [45]. The antioxidants in *L. leucocephala* suppress the microflora in the intestines of chickens [46]. FLM, starting at 4%, significantly increased egg production in the presence of FLM and improved intestinal performance in nutrient absorption by suppressing pathogenic microflora in the chicken gastrointestinal tract.

Egg production in this study was 54%–57% due to the weather conditions in Indonesia at the time of the study being hot, with temperatures between 32°C and 35°C and 80% humidity, so egg production was delayed. Egg production is affected by both genetic and environmental factors; higher temperatures significantly (p ≤ 0.05) delayed egg production [47]. FLM resulted in higher egg production (p ≤ 0.05) than without FLM. Egg production increased significantly with increasing FLM in feed, 2% FLM (54.54% ± 0.22%), 4% FLM (54.92% ± 0.18%), 6% FLM (55.25% ± 0.21%), and 8% FLM (56.43% ± 0.24%).

The feed conversion ratio (FCR) is a measure of feed efficiency [48]. The lower the FCR, the better the feed quality; a high FCR indicates poor feed quality. The FCR decreased significantly with the use of FLM, indicating improved feed efficiency. Feed intake has a strong influence on feed conversion. FLM treatment reduces conversion value, allowing feed ingredients to be used more efficiently and increasing egg production. The FCR in the diet without FLM had the highest value of 2.33 ± 0.32 (p ≤ 0.05), but the conversion value decreased with increasing use of FLM.

Egg weight is influenced by feed intake [49]. Diet protein content may be a determining factor for egg weight. Egg weight is influenced by the protein content consumed by poultry [50]. There is an increase in egg weight with increasing levels of FLM in the diet. FLM increased protein intake and thus egg weight. In addition to protein, calcium and phosphorus affect egg weight and egg production in quail [51]. FLM increased calcium and phosphorous intake, resulting in a denser shell matrix and influencing egg weight gain.

Shell thickness is a determinant of the external quality of eggs [52]. Shell thickness is influenced by the calcium and phosphor content of the feed consumed by the quail [53]. Calcium and phosphorous are normally obtained from the diet; an excess is retained in the bones, and a deficiency affects shell quality [54]. FLM increases the feed calcium content of the diet, and FLM increases calcium intake and phosphor intake, thereby increasing mineral absorption for deposition in the shell. Phosphorus and calcium content increase shell thickness [55]. The fermentation process increases the availability of phosphorus compounds [22].

The Haugh unit is a criterion used to determine egg quality and is measured based on albumen height and egg weight. Egg weight is calculated from the weight of egg components such as yolk, egg white, and shell [34, 54]. In addition, the quality of egg composition is determined by the fat and protein content of the diet [56]. Feeding FLM significantly affected egg composition and weight due to differences in protein intake and higher fat intake compared with the control. The addition of fat intake increased the proportions of white and yolk [57].

Our study showed that Haugh units were higher in the FLM diet than in the control diet because egg weight was higher in the FLM diet than in the control diet. The Haugh unit value is directly proportional to albumin height and egg weight [58–60]. When the egg weight is significantly different, the Haugh unit value also has a significant effect. The Haugh unit value of FLM is within the normal range [20, 60], whereas the diet without FLM is significantly lower than the normal Haugh unit [61].

The yolk color index is an indicator of egg yolk quality; the brighter the yolk color, the better the quality of the yolk. The yolk color index is influenced by feed intake. FLM containing carotenoid pigments such as lutein, zeaxanthin, and xanthophyll affect egg yolk color. There was an increase in the yolk color index with increasing FLM content in the diet. Higher levels of xanthophylls in animal feeds result in higher xanthophyll uptake by animals. Xanthophyll is stored in fatty tissues throughout the body and produces a yellowish color throughout the body, including egg yolks and xanthophyll [62, 63]. Egg production was lower than the standard at this age; however, other parameters showed a significant effect of FLM use compared with the control, which reduced feed conversion and improved egg quality, consisting of egg weight, shell thickness, Haugh units, and yolk color.

## CONCLUSION

This study demonstrates that incorporating FLM into the diet of laying quails effectively improves nutrient intake, egg production, and egg quality. FLM supplementation significantly increased energy, protein, fat, calcium, and phosphorus intake, with notable enhancements in egg production, eggshell thickness, Haugh unit, and yolk color, particularly at inclusion levels of 4%-8%. The reduction of FCR highlights the efficiency of FLM as a feed ingredient, while the unaffected fiber and overall feed intake suggest that FLM integration does not compromise the standard dietary intake.

The strength of this study lies in its novel approach of using fermentation to reduce the anti-nutritional factors in *Leucaena leucocephala*, enhancing its suitability as a protein source for poultry. The well-controlled experimental design and comprehensive analysis of production and quality metrics further substantiate the findings.

However, some limitations must be acknowledged. The study was conducted under controlled conditions, which may not fully replicate the variability in field conditions, such as environmental stressors or varying quail genotypes. In addition, the study focused primarily on laying quails, leaving scope to investigate the effects of FLM on other poultry species or at different production stages.

Future research should explore the long-term effect of FLM supplementation on quail performance and health. Further studies could also evaluate the economic feasibility of FLM production and its scalability for commercial poultry farming. Investigations into optimizing the fermentation process to maximize nutrient enhancement and reduce anti-nutritional factors further would contribute to the broader applicability of FLM as an alternative feed ingredient.

## AUTHORS’ CONTRIBUTIONS

MMDU: Conceptualized the study and drafted and revised the manuscript. AA: Conducted experiments and analyzed data. All authors have read and approved the final manuscript.

## References

[1] Lima H.J.D, Morais M.V.M, Pereira I.D.B (2023). Updates in research on Quail nutrition and feeding:A review. Worlds Poult. Sci. J.

[2] Mnisi C.M, Oyeagu C.E, Akuru E.A, Ruzvidzo O, Lewu F.B (2023). Sorghum, millet, and cassava as alternative dietary energy sources for sustainable Quail production:A review. Front. Anim. Sci.

[3] De Angelis A, Gasco L, Parisi G, Danieli P.P (2021). A multipurpose Leguminous plant for the Mediterranean countries:*Leucaena leucocephala* as an alternative protein source:A review. Animals (Basel).

[4] Abdelnour S.A, Abd El-Hack M.E, Ragni M (2018). The efficacy of high-protein tropical forages as alternative protein sources for chickens:A review. Agriculture.

[5] Tirajoh S, Dominanto G.P, Soplanit A, Bakrie B (2021). Production performance of KUB chicken with the inclusion of Lamtoro (*Leucaena leucocephala*) leaf flour in the feed. IOP Conf. Ser. Earth Environ. Sci.

[6] Zapata-Campos C.C, Garcia-Martinez J.E, Salinas-Chavir J, Ascacio-Valdés J.A, Medina-Morales M.A, Mellado M (2020). Chemical composition and nutritional value of leaves and pods of *Leucaena leucocephala*, *Prosopis laevigata*, and *Acacia farnesia* in a Xerophilus Shurbland. Emir. J. Food Agric.

[7] Sona K, Oematan G, Dato T, Mullik M.L (2023). Pengaruh level campuran daun lamtoro (*Leucaena leucocephala*) dan daun kelor (*Moringa oleifera*) terhadap berat, ukuran dan kandungan nutrisi maggot lalat tentara hitam (*Hermetia illucens*). Anim. Agric.

[8] Llanes J.A, Ramirez D.J (2022). Growth Performance and Carcass Characteristics of Broiler Chickens (*Gallus gallus domesticus*) Fed with Alternative Organic Feeds. [Master's Thesis], Ilocos Sur Polytechnic State College Graduate School.

[9] Kamel E.R, Mohammed L.S, Abdelfattah F.A.I (2020). Effect of a diet containing date pits on growth performance, diet digestibility, and economic evaluation of Japanese quail (*Coturnix coturnix japonica*). Trop. Anim. Health Prod.

[10] Afolafu S.A, Yusuf O.O, Abioye A.A, Emetere M.E, Ongbali S.O, Samuel O.D (2021). A sustainable renewable source of energy-a review. IOP Conf. Ser. Earth Environ. Sci.

[11] Jha R, Misha P (2021). Dietary fiber in poultry nutrition and their effects on nutrient utilization, performance, gut health, and on the environment:A review. J. Anim. Sci. Biotechnol.

[12] Tajeda J, Kim W (2021). Role of dietary fiber in poultry nutrition. Animals (Basel).

[13] Kaur G, Kumar A, Kurl S, Mittal N, Malik D.S, Bassi P, Singh T, Khan A.A, Alanasi A.M (2024). *Leucaena leucocephala* succinate based polyelectrolyte complexes for colon delivery of synbiotic in management of inflammatory bowel disease. Heliyon.

[14] Ramli N, Jamaludin A.A, Ilham Z (2017). Mimosine toxicity in *Leucaena* biomass:A hurdle impeding maximum use for bioproducts and bioenergy. Int. J. Environ. Sci. Nat. Res.

[15] Honda M.D.H (2021). The role of mimosine, a non-protein acid in *Leucaena leucocephala* subsp. Glabrata (*Giant leucaena*). PhD Dissertation.

[16] Liener I.E (2019). Antinutritional factors related to proteins and amino acids. In:Foodborne Disease Handbook.

[17] Abu Hafsa S.H, Hassan A.A, Elghandour M.M.M.Y, Barbarosa-Pliego A, Mellado M, Salem A.Z.M (2022). Dietary anti-nutritional factors and their roles in livestock nutrition. In sustainable Agriculture Review.

[18] Haetami K, Mukaromah F, Andhikawati A, Herman R.G (2023). The effect of giving Lamtoro meal (*Leucaena leucocephala*) with different concentrations in feed on the growth and survival rate of Tilapia (*Oreochromis niloticus*). Asian J. Fish. Aquat. Res.

[19] Wang Y, Wu J, Li L, Yao Y, Chen C, Hong Y, Chai Y, Liu W (2024). Effects of tannic acid supplementation of a high-carbohydrate diet on the growth, serum biochemical parameters, antioxidant capacity, digestive enzyme activity, liver, and intestinal health of largemouth bass, *Micropterus salmoides*. Aquac. Nutr.

[20] Alkhatib R, Mheidat M, Abdo N, Tadros M, Al-Eitan L, Al-Hadid K (2019). Effect of lead on the physiological, biochemical, and ultrastructural properties of *Leucaena leucocephala*. Plant Biol.

[21] Dai Z, Cui L, Li J, Wang B, Guo L, Wu Z, Zhu W, Wu G (2020). Fermentation techniques in feed production. In:Animal Agriculture.

[22] Nursiwi A, Arivia C.Y, Ishartani D, Sari A.M, Zaman M.Z (2024). Changes in chemical characteristics and dynamics population of microorganisms in pre-fermentation treatment of lamtoro (*Leucaena leucocephala*) during Mlanding Tempeh's production. Food Res.

[23] Şanlier N, Gökcen B.B, Sezgin A.C (2019). Health benefits of fermented foods. Crit. Rev. Food Sci. Nutr.

[24] Dewi E.R.S, Rahmawati F, Ulfah M (2022). The effect of EM-4 and ST probiotic fortification on the growth of Tilapia (*Oreochromis niloticus*) in the biofloc-aquaponic system. AACL Bioflux.

[25] Ramadani C.N, Andriyani Y, Suryadi I.B.B, Haetami K (2020). Effect of fermented lamtoro leaf (*Leucaena leucocephala*) meal on growth rate of giant gourami fingerlings (*Osphronemus gouramy*). Asian J. Fish. Aquat. Res.

[26] (2007). Commission Recommendation of 18 June 2007 on Guidelines for the Accommodation and Care of Animals Used for Experimental and Other Scientific Purposes (Notified under Document Number C (2007) 2525) (Text with EEA Relevance).

[27] Pratiwi S.H (2018). The effect of compost fertilizer and effective microorganism 4 doses on growth and yield of Pakcoy (*Brassica rapa* L.). Gontor Agrotech. Sci. J.

[28] Cahyani V.R, R, Lakshitarsari K.P, Megow R.A.Z.W, Azzahra N.Y (2024). Composting of rice straw-based materials using aerobic bioactivator isolated from rice straw, mahogany bark, and cassava peels. Caraka Tani J. Sustain. Agric.

[29] Joseph N.S, Robinson F.E, Korver D.R, Renema R.A (2000). Effect of dietary protein intake during the pullet-to-breeder transition period on early egg weight and production in broiler breeders. Poult. Sci.

[30] Varkoohi S, Babak M.M.S, Pakdel A, Javaremi A.N, Zaghari M, Kause A (2010). Response to selection for feed conversion ratio in Japanese Quail. Poult. Sci.

[31] Iqbal J, Khan S.H, Mukhtar N, Ahmed T, Pasha R.A (2016). Effects of egg size (weight) and age on hatching performance and chick quality of broiler breeder. J. Appl. Anim. Ethics Res.

[32] Fernandez I.B, Calixto L.F.L, Torres-Cordido K.A.A, Lemos M.J.D, Togashi C.K, Souza D.S.D, Alves O.D.S, Pizzolante C.C (2018). Feeding time under performance and eggs quality of Quails in production. Rev. Bras. Saúde Prod. Anim.

[33] Sabuncu M, Akdoğan M (2014). Utilizing optical coherence tomography in the nondestructive and noncontact measurement of eggshell thickness. Sci. World J.

[34] Haugh R.R (1937). The Haugh unit for measuring egg quality. US Egg Poult. Mag.

[35] Islam K.M, Khalil M, Männer K, Raila J, Rawel H, Zentek J, Schweigert F.J (2017). Lutein-specific relationship among spectrophotometric and colorimetric parameters of chicken egg yolk. J. Poult. Sci.

[36] Classen H.L (2017). Diet energy and feed intake in chickens. Anim. Feed Sci. Technol.

[37] Oboh G, Ademosun O, Lajide L (2012). Improvement of the nutritive value and antioxidant properties of Citrus peels through *Saccharomyces cerevisiae* solid substrate fermentation for utilization in livestock feed. Livest. Res. Rural Dev.

[38] Abdallah A.G, Beshara M.M, Ibrahim F (2015). Effect of different levels and sources of dietary fiber on productive and economic performance in local laying hens during the growing period and subsequent laying performance. Egypt. Poult. Sci.

[39] Putra A.N, Pradana A.C, Novriansyah D, Mustahal M (2019). Effect of dietary fermented Lamtoro (*Leucaena leucocephala*) leaves flour in feed on digestibility and hematological parameters of catfish (*Clarias sp*.). e-J. Rekayasa Teknol. Budidaya Perairan.

[40] Agbo A.N (2020). Effects of processing on amino acids composition of *Leucaena leucocephala* (Lam De Wit) leaf meal. Niger. J. Biotechnol.

[41] Morilla E.A, Taddia A, Sortino M, Tubio G (2023). Mixed cultures of *Aspergillus niger* and *Rhizopus oryzae* using lignocellulosic substrates to improve hydrolytic enzyme production. Bioenergy Res.

[42] Van Dung D, Shang W, Yao W (2014). Effect of crude protein levels in concentrate and concentrate levels in diet on *in vitro* fermentation. Asian-Australas. J. Anim. Sci.

[43] Barzegar S, Wu S.B, Choct M, Swick R.A (2020). Factors affecting energy metabolism and evaluating net of poultry feed. Poult. Sci.

[44] Artdita C.A, Zhuang Y.R, Liu T.Y, Cheng C.Y, Hsiao F.S.H, Lin Y.Y (2021). The effect of feeding restriction on the microbiota and metabolome response in late-phase laying hens. Animals (Basel).

[45] Kousalya P, Jayanthy V (2016). Evaluation of phytochemicals and quantification of phenol, flavonoids, and tannins of pods of *Leucaena leucocephala* (Lam.) De Wit. Am. Eurasian. J. Agric. Environ. Sci.

[46] Daeng I, Pathak A.K, Bhat A, Zargar A (2017). Antioxidant and antibacterial potential of condensed tannins containing tree leaves extract. Vet. Pract.

[47] Tůmová E, Gous R.M (2012). Interaction of hen production type, age, and temperature on laying pattern and egg quality. Poult. Sci.

[48] Yua J, Dou T, Ma M, Yi G, Qu L, Shen M, Qu L, Wang K, Yang N (2015). Genetic parameters of feed efficiency traits in laying period of chickens. Poult. Sci.

[49] Iqbal J, Mukhtar N, Rehman Z.U, Khan S.H, Ahmad T, Anjum M.S, Pasha R.H, Umar S (2017). Effects of egg weight on the egg quality, chick quality, and broiler performance at the later stages of production (Week 60) in broiler breeders. J. Appl. Poult. Res.

[50] Shim M.Y, Song E, Billard L, Aggrey S.E, Pesti G.M, Sodsee P (2013). Effects of balanced dietary protein levels on egg production and egg quality parameters of individual commercial layers. Poult. Sci.

[51] Stanquevis C.E, Furlan A.C, Marcato S.M, de Oliveira-Bruxel T.M, Perine T.P, Finco E.M, Grecco E.T, Benites M.I, Zancanela V (2021). Calcium and available phosphorus requirements of Japanese Quails in the early-laying stage. Poult. Sci.

[52] Samiullah S, Omar A.S, Roberts J, Chousalkar K (2017). Effect of production system and flock age on eggshell and egg internal quality measurements. Poult. Sci.

[53] Nys Y (2017). Laying hen nutrition:Optimizing hen performance and health, bone and eggshell quality. In:Achieving Sustainable Production of Eggs.

[54] Duman M, Şekeroğlu A, Yildirim A, Eleroğlu H, Camci Ö (2016). Relation between egg shape index and egg quality characteristics. Eur. Poult. Sci.

[55] Skrivan M, Englmaierova M, Marounek M, Skrivanova V, Taubner T, Vit T (2016). Effect of dietary magnesium, calcium, phosphorus, and limestone grain size on productive performance and eggshell quality of hens. Czech J. Anim. Sci.

[56] Kowalska E, Kucharska-Gaca J, Kuźniacka J, Lewko L, Gornowicz E, Biesek J, Adamski M (2021). Egg quality depending on the diet with different sources of protein and age of the hens. Sci. Rep.

[57] Grobas S, Mendez J, Lazaro R, De Blas C, Mateo G.G (2001). Influence of source and percentage of fat added to the diet on performance and fatty acid composition of egg yolks of two strains of laying hens. Poult. Sci.

[58] Dudosola I.O, Adeyemi E.A, Ayodele S.I (2019). Prediction of Japanese Quail egg weight using egg components as regressors. Niger. J. Anim. Prod.

[59] Mengsite F.W, Yitbarek M.B, Getachew E (2019). Productivity and egg quality traits of Sasso T44 chicken in North Showa Zone. Ethiop. J. Anim. Plant Sci.

[60] Northcutt J.K, Buyukyavuz A, Dawson P.L (2022). Quality of Japanese quail (*Coturnix coturnix japonica*) egg after extended refrigerated storage. J. Appl. Poult. Res.

[61] Kavtarashvili A.S, Stefanova I.L, Svitkin V.S (2019). Functional egg production, the role of the carotenoids. Agric. Biol.

[62] Zurak D, Slovenec P, Janječić Z, Bedeković X.D, Pintar J, Kljak K (2022). Overview on recent findings of nutritional and non-nutritional factors affecting egg yolk pigmentation. Worlds Poult. Sci. J.

[63] Zakariya A, Khamid M.N, Aryanti I, Wardi W (2024). The effect of Indigofera leaf meal on production performance and eggs quality of *Coturnix coturnix japonica*. J. Ilmu Peternakan Terapan.

